# The Future of Healthcare Is Digital: Unlocking the Potential of Mobile Health and E-Health Solutions

**DOI:** 10.3390/healthcare13070802

**Published:** 2025-04-03

**Authors:** Daniele Giansanti

**Affiliations:** Centro Tisp, Istituto Superiore di Sanità, 00161 Roma, Italy; daniele.giansanti@iss.it; Tel.: +39-06-49902701

In the era of rapid technological advancement, healthcare is undergoing a profound transformation driven by digital solutions. The integration of artificial intelligence (AI) and conversational agents, such as ChatGPT, is reshaping the way healthcare is delivered, offering innovative opportunities to enhance patient care, streamline workflows, and improve overall efficiency.

The Special Issue “Healthcare Goes Digital: Mobile Health and Electronic Health Technology in the 21st Century” [1] aimed to explore emerging themes, examining their innovative applications, challenges, and prospects. A crucial focus is on both telemedicine [2] and mobile health applications and their impact on healthcare delivery, patient monitoring, and disease management [3]. Another area of interest is wearable health technology [4] and its role in continuously monitoring health metrics, offering new possibilities for personalized medicine. The evolution and effectiveness of telemedicine, particularly with AI-driven diagnostic tools [5] and virtual consultations [6], also form a central part of the discussion today. Furthermore, the integration of AI-driven analytics in electronic health records (EHRs) [7] is crucial for clinical decision-making and interoperability.

Security and privacy concerns in digital healthcare [8], especially regarding AI-driven applications nowadays, are key considerations that must be faced to ensure the responsible deployment of these technologies. Patient engagement through digital platforms, including AI-driven chat interfaces and virtual health assistants, is another significant topic in rapid evolution [9]. Finally, the influence of AI, the Internet of Things (IoT) [10], and other emerging technologies in healthcare is a field in need of special attention, both for its potential and implications.

As digital healthcare continues to evolve, it is crucial to critically assess the potential benefits and challenges of the integration of all this emerging technology.

Thanks to the contribution of numerous international scholars, this Special Issue has collected, in addition to this editorial, 13 studies, including 7 scientific articles [11,12,13,14,15,16,17] and 6 reviews [18,19,20,21,22,23], 5 of which are systematic reviews [19,20,21,22,23].

*Contributions of the Article studies*

Table 1 provides a brief summary of the foci and contributions of the articles published in this Special Issue.

Some studies have explored the intersection of healthcare technology, patient care, and innovative solutions aimed at enhancing both accessibility and outcomes. One notable study by Alzghaibi [11], investigates barriers to the adoption of the Sehaty mobile health application, particularly for patients suffering from chronic diseases. It reveals challenges in technical performance, user interface design, and privacy concerns. This research provides valuable insights for improving mobile health platforms by enhancing stability, user experience, and security to ensure higher user satisfaction and engagement.

Lu et al. [12], delve into how online healthcare platforms influence patient decision-making. Specifically, their study looks at how hospital ratings and patient reviews shape the choices of patients seeking care across regions. The findings emphasize the need to optimize these systems to promote healthcare equity, thereby improving informed decision-making for underserved populations.

Shafran-Tikva et al. [13] propose a study taking a data-driven approach to preventing pressure injuries in elderly patients by analyzing electronic medical records. It identifies key factors that can help detect and prevent community-acquired pressure injuries, offering practical data that can improve clinical practices and patient safety.

In the field of patient-centered care, the work proposed by Estadella et al. [14] investigates the role of virtual reality (VR) in reducing pain and stress during medical procedures like office hysteroscopy. The study demonstrates that VR can significantly enhance patient comfort and reduce the need for invasive interventions, highlighting the potential of VR to revolutionize procedural care.

For mental health, Han et al. [15] explore the effects of virtual music therapy, based on positive psychology, on the mental health of college students during the COVID-19 pandemic. The findings underscore the effectiveness of such interventions in reducing stress, anxiety, and depression, especially during challenging times.

Artificial Intelligence (AI) also plays a crucial role in patient care, as highlighted by Gomez-Cabello et al. [16]. Their study evaluates the potential of AI models like ChatGPT-3.5 and GPT-4 in providing postoperative care advice to plastic surgery patients. It emphasizes the potential of large language models to deliver accurate and accessible care information, presenting them as valuable adjuncts in patient education.

Lastly, Lepri et al. [17] examine the shift toward home-based radiology services, particularly after the COVID-19 pandemic. The study uncovers the challenges and opportunities faced by medical radiology technicians, highlighting the need for further research and collaboration to integrate AI and improve patient care in a home setting.

Together, these studies shed light on the growing role of technology in enhancing patient care, from mobile health apps and AI integration to innovative pain management and virtual therapy solutions. The insights gained provide a foundation for the continued evolution of healthcare services, ensuring that they remain accessible, patient-centered, and efficient.

*Contribution of the review studies*

Table 2 focuses on the published review studies with a sketch. An overview of the reviews published in this Special Issue highlights the diverse ways in which digital interventions are transforming healthcare across various domains.

One of the most significant advancements is in telerehabilitation for chronic neck pain as highlighted by Valenza-Peña et al. [18]. Their review confirmed the efficacy of virtual consultations and remote exercise programs in reducing pain and improving functional outcomes for patients suffering from chronic neck pain. This demonstrates the growing potential of telerehabilitation to provide effective pain management and rehabilitation, particularly in remote or underserved areas.

Another key area of digital health innovation is the use of voice assistants (VAs) in managing non-communicable diseases (NCDs) as reported by Bramanti et al. [19]. The systematic review examining the role of VAs in managing illnesses like diabetes, cardiovascular diseases, and mental health conditions found that these technologies enhance patient engagement, improve self-management, and encourage behavioral changes. However, challenges such as privacy concerns and adoption barriers remain, which must be addressed to maximize their potential in healthcare settings.

In maternal and perinatal care, chatbots for women and expectant parents based on Amil et al. [20] have proven to be an invaluable resource. A systematic review of studies on the use of chatbots in supporting women throughout the reproductive cycle showed that these interactive tools significantly improved health knowledge, behaviors, and attitudes. They also facilitated better access to healthcare information and services, offering an effective way to engage expectant parents and women during preconception, pregnancy, and postpartum periods.

The use of digital psychotherapy in addressing suicide ideation and depression has also gained considerable attention as reported in Oh et al. [21]. This systematic review found that digital interventions, particularly Cognitive Behavioral Therapy (CBT), significantly reduced both suicide ideation and depression, providing a promising alternative to traditional face-to-face therapy. This approach offers greater accessibility and convenience, making it an increasingly important option for mental healthcare.

Bogár et al. [22], focused on the field of cardiac care, highlighting that smartwatches for arrhythmia detection [22] have shown to play a crucial role in the early detection and continuous monitoring of cardiac conditions such as atrial fibrillation. The systematic review highlights the potential of these wearable devices to enable timely interventions and improve patient outcomes, particularly for individuals at high risk of heart-related complications.

Lastly Protano et al. [23] demonstrated that digital technologies have also proven effective in promoting weight loss and healthy behaviors [23]. The systematic review of studies on mobile apps, wearables, and online programs for weight management demonstrated their effectiveness in encouraging healthier lifestyles, particularly by increasing physical activity and improving dietary habits. The personalized feedback provided by these digital tools has been shown to enhance weight loss efforts, offering significant benefits for individuals with obesity or overweight conditions.

Together, these reviews underline the transformative role of digital health technologies in modern healthcare. They highlight how virtual interventions, whether through telerehabilitation, voice assistants, chatbots, digital psychotherapy, or wearables, can enhance patient care, improve clinical outcomes, and provide accessible, personalized healthcare solutions across various health conditions.

*Conclusions and future routes*

Based on the contributions presented in this Special Issue, it is evident that healthcare technologies, including mobile health applications, virtual interventions, and digital platforms, are playing a transformative role in improving patient care, access, and overall health outcomes. The articles and reviews provide valuable insights into the challenges, opportunities, and effectiveness of these technologies across various healthcare domains, such as chronic disease management, mental health support, rehabilitation, and the monitoring of cardiovascular conditions [11,12,13,14,15,16,17].

Looking ahead, several key areas have been detected for further exploration and development. First, improving the usability, accessibility, and technical stability of mHealth platforms is crucial for ensuring their broader adoption and sustained engagement among patients, particularly those with chronic conditions [11]. In addition, focusing on the challenges related to data privacy, security, and the integration of digital tools into existing healthcare systems will be essential for maximizing their impact on patient care [12].

Future research should continue to focus on optimizing the integration of AI-powered tools, such as voice assistants, chatbots, and large language models, to enhance patient–provider communication, support self-management, and provide personalized care [19,20,21,22,23]. Moreover, the growing role of digital psychotherapy, telerehabilitation, and wearables in mental health and physical rehabilitation underscores the potential for remote healthcare interventions to complement traditional care models and offer more flexible, patient-centered solutions [18,21,22].

Lastly, as the healthcare landscape continues to evolve, it will be essential to address the ethical considerations surrounding the use of AI and digital technologies, ensuring that these tools are used responsibly and in ways that enhance health equity, patient autonomy, and trust in digital healthcare systems [19,23].

In conclusion, the ongoing advancement of digital health technologies promises to revolutionize healthcare delivery and provide more efficient, accessible, and personalized care. However, reaching the full potential of these technologies will require continued innovation, interdisciplinary collaboration, and a commitment to addressing the challenges that accompany their integration into real-world healthcare practices.

## Figures and Tables

**Table 1:** Sketch of the articles published in the Special Issue.

Study/Minititle	Focus	Brief Summary	Contribution
[11] Sehaty App Usability in Saudi Arabia	Mobile health (mHealth) app adoption and usability	This study investigates the barriers hindering the adoption of the Sehaty app among chronic disease patients in Saudi Arabia. It identifies issues like technical performance, navigation difficulties, privacy concerns, and accessibility challenges.	Provides actionable insights for improving the technical stability, user interface design, and security features of mHealth platforms to enhance user engagement and satisfaction.
[12] Cross-Regional Healthcare Choices	Online healthcare services and patient decision-making	The study examines how online medical platform signals (hospital ratings, patient reviews) influence patients’ decisions to seek cross-regional treatment. It explores how these signals impact healthcare choices in underserved regions.	Offers insights for improving online healthcare platforms by optimizing hospital ratings and review systems, promoting healthcare equity, and supporting informed decision-making.
[13] Community-Acquired Pressure Injuries (CAPIs) in Elderly	Data-driven detection and prevention of pressure injuries	The study analyzes the electronic medical records of elderly patients to identify key factors associated with community-acquired pressure injuries (CAPIs).	Highlights novel indicators that can help detect and prevent CAPIs in community care settings, providing valuable data for clinical practice and improving patient safety.
[14] Virtual Reality in Office Hysteroscopy	Use of VR for pain and stress management	This study evaluates the effectiveness of virtual reality (VR) in reducing pain and stress during office hysteroscopy procedures.	Demonstrates that VR can significantly reduce pain during medical procedures, especially in patients with lower baseline stress, contributing to less invasive, patient-centered care.
[15] Virtual Music Therapy for College Students	Mental health intervention using virtual music therapy	This study explores the effectiveness of a virtual music therapy program based on positive psychology to enhance mental health among college students during the COVID-19 pandemic.	Shows that positive psychology-based virtual music therapy can significantly reduce stress, anxiety, and depression in college students, especially during stressful times.
[16] Large Language Models in Postoperative Care	AI in patient care: Postoperative recommendations	The study compares the performance of LLMs (ChatGPT-3.5, GPT-4, Gemini) in providing postoperative care advice to plastic surgery patients.	Highlights the potential of LLMs in providing accurate, readable, and understandable postoperative care information, emphasizing their role as adjunct tools in patient care.
[17] Home Radiology Integration	Shifting landscape of diagnostic imaging with home radiology	The study examines the integration of home radiology into healthcare, especially post COVID-19. It explores the experiences and challenges of medical radiology technicians with domiciliary imaging.	Offers insights into the challenges and potential of home radiology, urging further research and collaboration to enhance patient-centric care

**Table 2:** Sketch of the review studies published in the Special Issue.

Study/Minititle (Type of Review)	Focus	Brief Summary	Contribution
[18] Telerehabilitation for Chronic Neck Pain (REVIEW)	Telerehabilitation interventions in managing chronic neck pain, particularly focusing on pain reduction and improving functional outcomes.	This systematic review and meta-analysis explore the effectiveness of telerehabilitation as a method for managing chronic neck pain, particularly through virtual consultations and remote exercise programs. It evaluates studies that address pain and disability reduction in patients.	The review confirms the efficacy of telerehabilitation in reducing pain and improving disability outcomes in patients with chronic neck pain. Remote interventions such as exercise programs and virtual consultations are highlighted as key contributors to positive outcomes.
[19] Voice Assistants in Non-Communicable Diseases (SYSTEMATIC REVIEW)	Investigating the role of voice assistants (VAs) in supporting the management of non-communicable diseases (NCDs) such as diabetes, cardiovascular diseases, and mental health conditions.	This systematic review analyzes studies on the use of voice assistants in managing NCDs. It looks at various aspects such as usability, acceptability, adherence, behavioral outcomes, and overall impact on clinical and quality-of-life outcomes for patients with chronic conditions.	The review emphasizes the potential of voice assistants to enhance patient engagement, improve self-management, and facilitate behavioral changes. However, it identifies challenges such as privacy concerns, speech recognition errors, and barriers to adoption that need to be addressed.
[20] Chatbots for Women and Expectant Parents (SYSTEMATIC REVIEW)	The use of interactive conversational agents (chatbots) in supporting women and expectant parents during the preconception, pregnancy, and postnatal periods.	This systematic review synthesizes studies on the application of chatbots in healthcare for women and their families, covering the entire reproductive cycle from preconception to 12 months postpartum. It focuses on chatbots’ impacts on health behaviors, knowledge, and service utilization.	The review demonstrates the positive impact of chatbots in improving health knowledge, behaviors, and attitudes, as well as facilitating better access to health information and interactions with healthcare providers during the perinatal period.
[21] Digital Psychotherapy for Suicide and Depression (SYSTEMATIC REVIEW)	The effectiveness of digital psychotherapy, particularly Cognitive Behavioral Therapy (CBT), in addressing suicide ideation and depression.	This study investigates the effects of digital psychotherapy on suicide ideation and depression, analyzing randomized controlled trials that compare digital interventions to traditional therapy. It provides a quantitative analysis of the impact on suicide and depression outcomes.	The findings suggest that digital psychotherapy has a significant positive effect on reducing suicide ideation and depression compared to traditional face-to-face therapy, making it a promising alternative for mental healthcare.
[22] Smartwatches for Arrhythmia Detection (SYSTEMATIC REVIEW)	The role of smartwatches in detecting and monitoring cardiac arrhythmias, especially atrial fibrillation, and their potential integration into clinical care.	This systematic review gathers evidence on the use of smartwatches for arrhythmia detection, focusing on their ability to monitor heart conditions like atrial fibrillation. It examines various case studies and cohort studies on smartwatch-based arrhythmia detection.	The review highlights the potential of smartwatches as a tool for the early detection and continuous monitoring of arrhythmias, offering the possibility for timely interventions and more effective patient care, particularly for those at risk of heart-related complications.
[23] Digital Technologies for Weight Loss (SYSTEMATIC REVIEW)	Evaluating the effectiveness of digital interventions (such as mobile apps, wearables, and online programs) in promoting weight loss and improving lifestyle behaviors related to obesity.	This systematic review investigates digital interventions aimed at promoting weight loss in individuals with overweight or obesity. It includes studies that employ mobile technologies to increase physical activity and improve dietary habits, focusing on their impact on weight management.	The review concludes that digital technologies, particularly those offering personalized feedback, are effective in promoting weight loss and encouraging healthy behaviors in individuals with overweight or obesity, enhancing the overall effectiveness of lifestyle interventions.
